# The Effectiveness of High-Flow Nasal Cannula in Respiratory Distress in the Pediatric Population of Bundelkhand Region: An Observational Study

**DOI:** 10.7759/cureus.94317

**Published:** 2025-10-10

**Authors:** Om S Chaurasiya, Kawalpreet Chhabra, Akanksha Singh

**Affiliations:** 1 Pediatrics, Maharani Laxmi Bai Medical College, Jhansi, IND

**Keywords:** bronchiolitis, bronchopneumonia`, high-flow nasal cannula, mechanical ventilator, respiratory distress

## Abstract

Introduction: Respiratory distress is the most frequently encountered cause of admission in the pediatric intensive care unit (PICU) requiring immediate medical attention. Standard oxygen therapy is provided via traditional nasal cannula, oxygen mask, or oxygen hood, whose failure warrants the need for invasive mechanical ventilation. High-flow nasal cannula (HFNC) is increasingly being used as a form of noninvasive respiratory support with fewer side effects and has been shown to reduce the requirement for intubation.

Objectives: To evaluate the effectiveness of HFNC in various indications of respiratory distress and its effect on the length of hospital stay and further complications associated with it.

Method: We conducted a prospective observational study at our tertiary PICU between May 2023 and April 2024. Patients aged one month to 18 years with respiratory distress were initially administered standard oxygen therapy escalating to HFNC upon failure. The clinical data and investigations were reviewed.

Result: Among 100 pediatric patients presenting with respiratory distress, 53 were males, with a male-to-female ratio of 1.13:1, and a median age of two years, ranging from three months to 15 years. The most common indication for HFNC was bronchopneumonia (44, 44%), bronchiolitis (31, 31%), asthma (8, 8%), wheeze-associated lower respiratory tract infections (WALRI) (7, 7%), post-extubation (7, 7%), and congenital heart disease with respiratory distress (CHD) without congestive heart failure (CHF) (3, 3%). The overall success rate of HFNC therapy was 87 (87%). The average duration of stay among the success group was 8.69 + -2.26 days, and the failure group was 10.00 + -3.44 days. The average duration of HFNC was markedly longer in the success group (59.39 + -19.84 days) as compared to the failure group (5.69 + -2.02 hours). Factors associated with HFNC failure and the need for invasive ventilation included lower initial and minimum respiratory rate-oxygenation (ROX) indices, as well as higher initial and maximum fraction of inspired oxygen levels.

Conclusion: HFNC can be employed as the first line of noninvasive oxygen therapy in cases of respiratory distress, offering an efficient and compassionate alternative that may significantly reduce the incidence of mechanical ventilation.

## Introduction

Acute respiratory distress is a leading cause of pediatric intensive care unit (PICU) admissions, manifesting as increased respiratory rate (RR) and heart rate (HR), nasal flaring, hypoxemia, and the use of accessory muscles. Common conditions causing respiratory distress in children include acute bronchiolitis, wheeze-associated respiratory infections (WALRI), asthma, pneumonia, and congenital heart disease. Oxygen therapy is the primary treatment for these conditions and can be administered noninvasively through methods like nasal cannula or high-flow nasal cannula (HFNC), or invasively via mechanical ventilation. HFNC offers several advantages over traditional oxygen delivery methods by providing heated, humidified oxygen at flow rates that meet or exceed patients' inspiratory demands, as discussed by Mikalsen et al. [1]. This approach helps maintain functional residual capacity, reduce airway resistance, and improve overall oxygenation while minimizing the discomfort associated with other oxygenation methods. Thus, HFNC can be utilized as a cost-effective and economically affordable method of supplementing standard oxygen therapy, especially in resource and expertise-limited settings.

The previous studies conducted have not been able to elucidate the specific indications of HFNC and its impact on patient outcomes, and the circumstances under which it may not be suitable or effective. Hence, this study rigorously evaluates the efficacy of HFNC in managing respiratory distress in patients aged between one month and 18 years. This research also aims to offer a comprehensive assessment of HFNC's impact on pediatric respiratory conditions and address existing gaps in the literature.

Aim

The study was conducted to identify indications and the effectiveness of HFNC in respiratory distress in the pediatric population. The study also elucidated the reasons for treatment failure and complications regarding the use of HFNC. It also assessed the duration of oxygen therapy and the percentage of patients requiring mechanical ventilation after HFNC use and compared the duration of oxygen therapy, HFNC usage, and hospital stay between the success and failure groups.

## Materials and methods

It was a prospective observational study, which included 100 patients aged between one month and 18 years who presented with signs and symptoms of respiratory distress and gave informed consent. The exclusion criteria included children with congenital and acquired diseases in congestive cardiac failure, chronic kidney diseases with fluid overload states, pneumothorax, severe neutropenia, hemodynamically unstable shock, and a history of long-term ventilator dependency. Patients with respiratory distress fulfilling the inclusion criteria for HFNC were included in the study. The demographic data, including age, gender, socioeconomic status (SES), weight, clinical characteristics with relevant previous medical history, chief complaints, and diagnosis, were collected. The patient was initially administered standard oxygen therapy; following failure of which, the patient was put on an HFNC machine functioning as a heated humidified HFNC. The temperature was set to 34℃. The initial flow rate depended upon the age and weight of the patient. Respiratory variables were recorded, and arterial blood gas analyses were conducted before admission to the PICU as baseline and subsequently at one hour, six hours, and 24 hours after initiation of HFNC therapy in the PICU. The fraction of inspired oxygen (FiO2) was adjusted to reach a pulse oximetry (SpO2) between 92 and 97%. Failure of HFNC was considered when the patient’s respiratory condition did not improve or worsen, and the patient needed escalation to a noninvasive mode of ventilation or intubation in the intensive care unit. The primary outcome was recorded as a change in SpO2 after HFNC application at one hour, six hours, and 24 hours. The secondary outcomes included changes in arterial blood gas, vital parameters, and oxygenation variables.

The data was analyzed using a recent version of the IBM SPSS Statistics for Windows, Version 24 (Released 2016; IBM Corp., Armonk, New York, United States). Chi-square test was used to test the significance of the difference between quantitative variables and Yates' test for qualitative variables.

## Results

The result of our prospective observational study conducted on a sample size of 100 patients found that younger age group patients between six months and two years have a higher incidence of respiratory distress. The primary indications for initiating HFNC were bronchopneumonia (44, 44%), bronchiolitis (31, 31%), asthma (8, 8%), post-extubation (7, 7%), and WALRI (7, 7%). The overall success rate of HFNC in our study was 87% with higher success observed in cases of asthma (8, 100%), WALRI (7, 100%), followed by bronchiolitis (27, 87.1%), post-extubation (6, 85.7%), bronchopneumonia (37, 84.1%), and congenital heart disease with respiratory distress (CHD) without congestive heart failure (CHF) (2, 66.7%) (Table 1).

**Table 1:** Demographic characteristics, underlying medical conditions and primary indications of study participants CHD with RD: congenital heart disease with respiratory distress; CHF: congestive heart failure; HFNC: high-flow nasal cannula; WALRI: wheeze-associated lower respiratory tract infections; SES: socioeconomic status

Characteristics	Total	Success (n = 87)	Failure (n = 13)	p-value	
					Age	-	-	-	0.020*	
1 m-6 m	16	10 (11.5)	6 (46.2)	-	
6 m-2 y	41	36 (41.4)	5 (38.4)	-	
2 y-5 y	24	23 (26.4)	1 (7.7)	-	
5 y-10 y	10	10 (11.5)	0 (0.0)	-	
10 y-18 y	9	8 (9.2)	1 (7.7)	-	
Gender	-	-	-	0.948	
Male	53	46 (52.9)	7 (53.8)	-	
Female	47	41 (47.1)	6 (46.2)	-	
SES	-	-	-	0.575	
Class 3	15	14 (16.1)	1 (7.6)	-	
Class 4	50	44 (50.6)	6 (46.2)	-	
Class 5	35	29 (33.3)	6(46.2)	-	
Primary indication for HFNC	-	-	-	-	
Bronchiolitis	31	27 (87.1)	4 (12.9)	0.985	
Bronchopneumonia	44	37 (84.1)	7 (15.9)	0.443	
Asthma	8	8 (100.0)	0 (0.0)	0.254	
Post-extubation	7	6 (85.7)	1 (14.3)	0.916	
CHD with RD without CHF	3	2 (66.7)	1 (33.3)	0.288	
WALRI	7	7 (100.0)	0 (0.0)	0.289	

Improvements in HR, RR, pulse oximetry, and SpO2/FiO2 ratio, along with increasing ROX index, were observed after the initiation of HFNC, which led to significant changes in the level of hypoxemia and work of breathing. The improvement in blood gas parameters involving pH, PaO2, and PaCO2, especially lactate levels, was observed in the success group (Table 2).

**Table 2:** Evolution of clinical parameters ROX index: respiratory rate-oxygenation index; HFNC: high-flow nasal cannula; HR: heart rate; RR: respiratory rate; SpO2: oxygen saturation; FiO2: fraction of inspired oxygen; PaCO2: partial pressure of arterial carbon dioxide

Clinical parameters	Baseline (before HFNC)	Early HFNC period (0.5-8 h)	p-value	Late HFNC period (8-24 h)	p-value	
						HR (beats/min)	103.43 ± 13.58	98.22 ± 11.38	<0.001*	94.80 ± 7.86	<0.001*	
RR (breaths/min)	52.64 ± 14.90	49.35 ± 14.06	<0.001*	43.66 ± 12.13	<0.001*	
SpO2 (%)	80.26 ± 7.50	93.92 ± 2.24	<0.001*	96.31 ± 1.21	<0.001*	
SpO2/FiO2 ratio	239.48 ± 80.27	171.47 ± 33.9	<0.001*	208.33 ± 38.41	<0.001*	
ROX index	4.81 ± 1.89	3.75 ± 1.21	<0.001*	5.07 ± 1.46	0.525	
Venous blood gas parameters	-	-	-	-	-	
pH	7.19 ± 0.15	7.30 ± 0.11	<0.001*	7.36 ± 0.76	<0.001*	
PaCO2	40.19 ± 6.88	37.92 ± 7.05	<0.001*	36.55 ± 4.51	<0.001*	
Lactate	3.03 ± 2.01	2.15 ± 1.12	<0.001*	1.50 ± 0.90	<0.001*	

The average duration of hospital stay among the successful group was (8.69 ± 2.26 days), and the failure group was (10.00 ± 3.44 days), respectively. For the given data, p < 0.05 was considered statistically significant (Table 3).

**Table 3:** Details of HFNC use according to diagnostic indication HFNC: high-flow nasal cannula; LOS: length of stay; CHD with RD: congenital heart disease with respiratory distress; CHF: congestive heart failure; WALRI: wheeze-associated lower respiratory tract infections; FiO2: fraction of inspired oxygen

Primary indication for HFNC	Total	HFNC duration (hours)	Peak FiO_2_ (%)	Peak flow		Hospital LOS
				L/min	L/kg	
Bronchiolitis	31	43.90 ± 20.26	56.94 ± 8.23	16.64 ± 4.80	2.78 ± 0.38	7.42 ± 1.71
Bronchopneumonia	44	57.48 ± 30.28	67.04 ± 7.34	39.14 ± 23.34	2.53 ± 0.39	8.86 ± 2.57
Asthma	8	57.50 ± 18.93	55.00 ± 7.56	53.75 ± 14.82	2.45 ± 0.37	8.38 ± 2.33
Post-extubation	7	54.29 ± 28.85	68.57 ± 3.78	51.14 ± 30.45	2.95 ± 0.34	13.57 ± 1.51
CHD with RD without CHF	3	44.67 ± 36.29	66.67 ± 5.77	17.33 ± 6.81	2.83 ± 0.29	8.00 ± 2.00
WALRI	7	53.86 ± 11.69	54.28 ± 7.87	46.71 ± 12.99	2.47 ± 0.31	7.71 ±0.49

Among the failure group (13, 13%), lower initial and minimum SpO2/FiO2 ratio as well as lower initial and minimum ROX index were observed (Table 4).

**Table 4:** Monitoring and final outcome according to primary indication of HFNC HFNC: high-flow nasal cannula; CHD: coronary heart disease; RD: respiratory distress; CHF: congestive heart failure; WALRI: wheeze-associated lower respiratory tract infections; ROX index: respiratory rate-oxygenation index; SpO2: oxygen saturation; FiO2: fraction of inspired oxygen

Primary indication for HFNC	Total	SpO2/FiO2 ratio			ROX index			Final outcome	
		Initial	Lowest		Initial	Lowest		Success (%)	Failure (%)
Bronchiolitis	31	300.40 ± 55.87		163.43 ± 22.95	4.88 ± 1.58		2.72 ± 0.72	27 (87.1)	4 (12.9)
Bronchopneumonia	44	217.04 ± 80.78		137.19 ± 16.88	4.94 ± 2.17		3.18 ± 0.91	37 (84.1)	7 (15.9)
Asthma	8	223.08 ± 39.90		171.80 ± 22.97	5.31 ± 1.54		4.08 ± 0.49	8 (100.0)	0 (0.0)
Post-extubation	7	161.43 ± 47.40		133.74 ± 9.20	3.61 ± 1.72		2.87 ± 0.66	6 (85.7)	1 (14.3)
CHD with RD without CHF	3	242.22 ± 54.30		133.8 ± 15.14	3.54 ± 1.12		1.98 ± 0.24	2 (66.7)	1 (33.3)
WALRI	7	206.31 ± 84.98		169.55 ± 20.55	4.90 ± 1.90		4.09 ± 0.64	7 (100.0)	0 (0.0)

## Discussion

This was a prospective observational study conducted on 100 patients. The majority of the participants in this study were aged between six months and two years, consistent with prior research that identifies this age group as highly susceptible to conditions like bronchiolitis and bronchopneumonia, which are common causes of respiratory distress in children. This demographic profile is similar to the cohorts studied by McKiernan et al., Franklin et al., and Wing et al., who also noted that younger children are more prone to requiring HFNC therapy due to the severity of these respiratory conditions [2-4]. The study also noted a slight male predominance of 53 (53%), which aligns with findings from Joshi et al., suggesting that boys may have a higher incidence of pediatric respiratory distress, but this may also be attributed to community bias toward a particular gender [5]. However, as Saslow et al. observed, gender itself does not significantly influence the outcomes of HFNC therapy, with other factors such as age and severity of illness playing more critical roles in determining the effectiveness of treatment [6].

The study demonstrated a high success rate of 87 (87%) for HFNC therapy, which is consistent with the success rates reported in previous studies. For instance, Milési et al. and Franklin et al. reported similar success rates of around 82% to 86%, particularly in cases of bronchiolitis and asthma [7,3]. The success of HFNC in these cases is likely due to its ability to provide heated, humidified oxygen at flow rates that meet or exceed the patient's inspiratory demand, thereby improving oxygenation and reducing the work of breathing. The higher failure rates observed in infants aged one to six months (6, 46.2%) are consistent with other studies, such as those by Kubicka et al., which also highlighted the challenges of managing respiratory distress in this vulnerable age group that could be attributed to smaller airway sizes, increased severity of disease, and a higher likelihood of complications like nasal obstruction or air leaks [8].

The incidence of complications in this study was 11%, with the most common issue being abdominal distension (5, 45.4%), nasal injury (4, 36.4%), and nasal crusting (2, 18.2%). The complications were managed according to standard protocols. These findings are in line with previous reports by Milési et al. and Kelly et al., who documented similar complication rates [7,9]. While these complications were relatively minor and manageable, they highlight the need for careful monitoring and prompt intervention to prevent more severe outcomes. The overall safety profile of HFNC remains favorable, with 89 (89%) of the patients not experiencing any complications, reaffirming its role as a reliable and effective noninvasive respiratory support modality.

In our study, the association between underlying medical conditions and outcomes of HFNC respiratory support was explored in pediatric patients. Patients with more complex medical conditions, such as CHD or a history of NICU admission for respiratory distress, had a higher failure rate of 20%, suggesting that these conditions may complicate HFNC therapy although this differ with previous studies, such as those by Franklin et al. and Hough et al. where no significant association was present between conditions like asthma or CHD and HFNC outcomes [3,10].

The study identified that higher initial and lowest SpO2/FiO2 ratios, as well as higher ROX indices, were significantly associated with successful outcomes. Patients who succeeded with HFNC had an initial SpO2/FiO2 ratio of 251.98 ± 73.9, while those who failed had a much lower ratio of 155.79 ± 72.6 (p < 0.001), which was statistically significant. These findings align with those of Roca et al. and Chisti et al., who demonstrated that higher initial oxygenation and ROX index values were strong predictors of positive outcomes in HFNC therapy [11,12]. Similarly, the initial and maximum FiO2 levels were also significant predictors of failure. The failure group required significantly higher initial (70.00 ± 7.07%) and maximum (73.84 ± 6.50%) FiO2 compared to the success group (59.83 ± 8.30% and 60.40 ± 8.22%, respectively), indicating that higher oxygen requirements are linked to more severe respiratory distress and a higher likelihood of HFNC failure. Similar results are quoted in Chang et al., where the failure rate was 15.7% (16 of 102 children) [13]. These findings emphasize the importance of early and continuous assessment of oxygenation parameters to guide clinical decisions regarding the continuation or escalation of respiratory support.

The duration of HFNC therapy was significantly longer in the success group, averaging 59.39 ± 19.84 hours, compared to just 5.69 ± 2.02 hours in the failure group (p < 0.001). This suggests that patients who respond positively to HFNC tend to stabilize over a longer period, allowing for continued noninvasive support rather than escalating to more invasive measures like mechanical ventilation. Conversely, the shorter duration of HFNC in the failure group may indicate the early identification of nonresponders, prompting quicker transition to alternative treatments. These findings align with previous studies by Franklin et al., who reported that prolonged HFNC usage might reflect a more stable clinical course, allowing patients to benefit from non-invasive respiratory support over extended periods [3]. Mayfield et al. recognized that nonresponders to HFNC can be identified early with a four times higher risk of admission to PICU in the standard treatment group than in the HFNC group [14].

Interestingly, the length of hospital stay (LOS) did not significantly differ between the success and failure groups, with the success group having a mean LOS of 8.69 ± 2.26 days compared to 10.00 ± 3.44 days in the failure group (p = 0.574). However, the lack of a significant difference in this study may reflect the severity of illness in the failure group or the prompt escalation to mechanical ventilation, which could have mitigated the expected increase in LOS.

The significant improvements observed in clinical parameters, such as HR, RR, and oxygen saturation (SpO2), further validate the efficacy of HFNC therapy. Kallappa et al. surveyed that a decrease in HR, RR, and improvement in pH, PaCO2 occurred within four hours of initiating HFNC with no adverse events [15]. The gradual improvements in these vital parameters reflect HFNC's effectiveness in enhancing respiratory function, reducing the workload on the cardiovascular system, and improving oxygenation, which are essential for patient recovery.

The SpO2 improvement from 80.26 ± 7.50% at baseline to 96.31 ± 1.21% in the late HFNC period highlights the therapy's effectiveness in enhancing oxygenation. The significant increase in pH from 7.19 ± 0.15 at baseline to 7.36 ± 0.76 during the late HFNC period, coupled with a decrease in PaCO2 from 40.19 ± 6.88 mmHg to 36.55 ± 4.51 mmHg, reflects improved ventilation and reduced hypercapnia, key indicators of enhanced respiratory status. Additionally, the decrease in serum lactate levels from 3.03 ± 2.01 mmol/L at baseline to 1.50 ± 0.90 mmol/L in the late HFNC period suggests a reduction in metabolic stress, further supporting the therapy's role in improving overall physiological stability. These improvements are consistent with findings like Mikalsen et al., who reported significant enhancements in vital signs and blood gas parameters during HFNC therapy [1]. Kelly et al. reported that failure occurred in more critical children who presented to the pediatrics emergency department with a triage respiratory rate greater than the 90th percentile for age, initial venous PCO2 of >50 mm Hg, and pH of <7.30 (significant respiratory acidosis) [9]. The high success rate, coupled with improvements in key clinical and blood gas parameters, suggests that HFNC should be considered as a first-line noninvasive respiratory support in PICU.

The study is limited as it was conducted at a single center and without a control group, which makes us cautious in generalizing the findings to other populations or healthcare environments. The sample size is not large enough to detect more subtle differences in outcomes among subgroups of patients with different underlying conditions or varying severities of illness. Given the observational nature of the study, other interventions or treatments could have also influenced the outcomes, which were not controlled in the analysis. The study does not compare other therapies, which limits conclusions about its relative efficacy and generalizability.

## Conclusions

HFNC may be considered as an effective first-line noninvasive oxygen therapy in pediatric patients with respiratory distress. Minimal side effects and cost-effectiveness, especially in resource-limited settings, add to its benefits. It also abates the use of invasive mechanical ventilation, thus decreasing hospital stay and morbidity.
